# From Increased Fluid Shear Stress to Natural Bypass Growth

**DOI:** 10.3390/ijms21103707

**Published:** 2020-05-25

**Authors:** Elisabeth Deindl, Paul H. A. Quax

**Affiliations:** 1 Walter-Brendel-Centre of Experimental Medicine, University Hospital, Ludwig-Maximilians-University, 81377 Munich, Germany; 2 Department of Surgery, Einthoven Laboratory for Experimental Vascular Medicine, Leiden University Medical Center, 2300 RC Leiden, The Netherlands

This Special Issue enqueues a series of publications dealing with arteriogenesis, which is the growth of a natural bypass from pre-existing arteriolar connections, as defined by Wolfgang Schaper, Werner Risau and Ramon Munoz-Chapuli in the late nineties of the last century. In times of increasing numbers of patients with cardiovascular occlusive diseases not only in highly industrialized but in almost all countries of the world, it is of major importance to understand the molecular mechanisms of this tissue- and life-saving process, which was given to us by mother nature to compensate for the function of a stenosed coronary or peripheral artery non-invasively. Since our first investigations on collateral artery growth more than 20 years ago, a lot of progress has been made, which we try to give access to in this issue on arteriogenesis. 

In the current special issue, entitled “Arteriogenesis – Molecular Regulation, Pathophysiology and Therapeutics II“ in the *International Journal of Molecular Sciences*, Zeen Aref and Margreet de Vries, from the group around Paul Quax, give an overview of the currently available variations of murine hind limb models for the study of angiogenesis and arteriogenesis, and highlight their advantages and disadvantages [1]. Huan Zhang and Don Chalothron, from Jim Fabers´ group, describe the features of collateral vessels and supply insights into the unique phenotypes and features of collateral endothelial and smooth muscle cells [2].

The trigger for arteriogenesis is increased fluid shear stress, which is exerted on the endothelial cells of pre-existing collateral arteries by blood flow being redirected around stenosed vessels. For a long time, it was completely unknown how this increased mechanical stress results in local leukocyte recruitment promoting collateral artery growth. Co-workers of the group of Elisabeth Deindl describe the functional role of extracellular RNA in that process and highlight the role of this nucleic acid during ongoing arteriogenesis [3]. Moreover, the same group presents a simple flow cytometry-based method to identify and quantify tissue infiltrated leukocyte subpopulations [4]. Macrophages, which accumulate in the perivascular tissue of growing collateral arteries, are well described for their pro-arteriogenic feature. Co-workers of Kerstin and Christian Troidl delineate the relevance of alternatively and classically activated macrophages, and explain the function of IL10 in that context [5].

Arterial occlusion results in reduced perfusion, and hence ischemia, in distally located tissue. As a consequence of the thereto related hypoxia, angiogenesis is induced. In contrast to other ischemic conditions, where capillaries are required to supply tissue locally with oxygen and nutrients, capillaries in regions distal to occluded arteries are necessary for the removal of cell debris. Yvonn Heun and the group around Hanna Mannel show that the tyrosine phosphatase SHP-2 inhibits 26S proteasome and thereby activates hypoxia-induced HIF-1α (hypoxia inducible factor 1α). This mechanism, which was identified in wounds, seems to be generally applicable, and results in pro-angiogenic gene expression under hypoxic conditions in ischemic tissue [6].

The process of collateral artery growth is often severely compromised in patients with diabetes mellitus. Srinivasan Vedantham and Elisabeth Deindl investigated the role of insulin for arteriogenesis in diabetic mice. They were able to attribute to this peptide hormone a function in the expression of Egr-1 (early growth response-1), a transcription factor relevant for collateral outward remodeling [7]. Cathrine Gorick, John Chappell and Richard Price explain the technical possibilities of ultrasound together with microbubbles as a non-invasive and spatially targeted option to therapeutically stimulate revascularization and point to perspectives of such approaches [8].

Thomas-Schmitz-Rixen, Kerstin Troidl and co-workers have developed an exercise training protocol in mice allowing the investigation of the effect of this physical force on collateral artery growth in mice [9], and, together with co-workers of Winfried Banzer, they show the effect of resistance training with and without blood flow restriction on the expression of circulating micro RNAs, which are relevant for collateral artery growth, in healthy volunteers [10]. 

Last but not least, the interventional cardiologists and specialists in the field of arteriogenesis, Bigler Marius Reto and Christian Seiler, provide an overview of human coronary collateral circulation and introduce the different clinical therapeutic approaches for the promotion of arteriogenesis in patients. They explain the problematic nature of biochemical concepts involving, for example, G-CSF (granulocyte-colony stimulating factor) and GM-CSF (granulocyte-macrophage colony stimulating factor), review the feasibility of biophysical concepts such as physical exercise and ECP (external counterpulsation), and focus on the promising approach of permanent occlusion of the internal mammary arteries in promoting natural bypass growth in patients [11]. 

With all our investigations and efforts, we think that we have come much closer to the goal of all of us to understand the mechanisms of collateral artery growth, finally enabling clinicians to promote arteriogenesis effectively in patients with vascular occlusive diseases. 
